# The Association between Short-Term Exposure to PM_1_ and Daily Hospital Admission and Related Expenditures in Beijing

**DOI:** 10.3390/toxics12060393

**Published:** 2024-05-28

**Authors:** Jingwen Xu, Yan Chen, Feng Lu, Lili Chen, Zhaomin Dong

**Affiliations:** 1 School of Cancer and Pharmaceutical Sciences, Faculty of Life Sciences and Medicine, King’s College London, London SE1 1UL, UK; 2 Ganzhou People’s Hospital, Ganzhou 341000, China; 3 Beijing Municipal Health Big Data and Policy Research Center, Beijing 100034, China; 4 School of Public Health, Southeast University, Nanjing 210009, China; 5 School of Materials Science and Engineering, Beihang University, Beijing 100191, China

**Keywords:** PM_1_, hospital admission, hospital expenditures, COPD, Beijing

## Abstract

Ambient particulate matter (PM) pollution is a leading environmental health threat worldwide. PM with an aerodynamic diameter ≤ 1.0 μm, also known as PM_1_, has been implicated in the morbidity and mortality of several cardiorespiratory and cerebrovascular diseases. However, previous studies have mostly focused on analyzing fine PM (PM_2.5_) associated with disease metrics, such as emergency department visits and mortality, rather than ultrafine PM, including PM_1_. This study aimed to evaluate the association between short-term PM_1_ exposure and hospital admissions (HAs) for all-cause diseases, chronic obstructive pulmonary disease (COPD), and respiratory infections (RIs), as well as the associated expenditures, using Beijing as a case study. Here, based on air pollution and hospital admission data in Beijing from 2015 to 2017, we performed a time-series analysis and meta-analysis. It was found that a 10 μg/m^3^ increase in the PM_1_ concentration significantly increased all-cause disease HAs by 0.07% (95% Confidence Interval (CI): [0, 0.14%]) in Beijing between 2015 and 2017, while the COPD and RI-related HAs were not significantly associated with short-term PM_1_ exposure. Meanwhile, we estimated the attributable number of HAs and hospital expenditures related to all-cause diseases. This study revealed that an average of 6644 (95% CI: [351, 12,917]) cases of HAs were attributable to ambient PM_1_, which was estimated to be associated with a 106 million CNY increase in hospital expenditure annually (95% CI: [5.6, 207]), accounting for 0.32% (95% CI: [0.02, 0.62%]) of the annual total expenses. The findings reported here highlight the underlying impact of ambient PM pollution on health risks and economic burden to society and indicate the need for further policy actions on public health.

## 1. Introduction

Ambient particulate matter (PM) pollution has been one of the of the leading environmental health threats in the world, especially in developing countries [1,2,3]. Existing epidemiological studies have associated ambient exposure to PM_10_ (PM with an aerodynamic diameter of ≤10 μm), PM_2.5_ (fine PM with aerodynamic diameter ≤ 2.5 μm), and PM_1_ (PM with an aerodynamic diameter of ≤1.0 μm) with morbidity and mortality of conditions such as cardiorespiratory and cerebrovascular diseases [1,4,5]. As reported, the global attributable mortality associated with air pollution, including ambient PM exposure, accounted for approximately 2.92 million female deaths and 3.75 million male deaths. Asian countries, including China, remain the regions with the highest health risk associated with air pollution [6]. In particular, low- and middle-income countries account for 62.6% of the global burden of pulmonary diseases, including chronic obstructive pulmonary disease (COPD) and lung cancer [7]. This evidence therefore indicates an urgent need to understand the underlying causes of PM-associated health risk and provides guidance for public health officials regarding air pollution control.

Previous studies have mostly focused on the effects of short-term exposure of PM_2.5_ and PM_10_, with various morbidity and mortality outcomes [8,9,10], while recent studies have pointed out that smaller sizes, such as PM_1_, may trigger higher risk. In most areas of China, PM_1_ concentrations are typically low, except in regions such as the North China Plain and Sichuan Basin, where intense human activities and unfavorable natural conditions are prominent, particularly during the winter months [11]. Meanwhile, the ratios of PM_1_ to PM_2.5_ ranged from 0.75 to 0.88, with higher values observed in January and lower values in August [12]. Regarding spatial distribution, regions such as North-Eastern China, the North China Plain, coastal areas of Eastern China, and the Sichuan Basin exhibited higher PM_1_/PM_2.5_ ratios (>0.9). In contrast, remote areas in Northwestern and Northern China, including Xinjiang, Tibet, and Inner Mongolia, demonstrated lower ratios (<0.7) [12]. In the North China Plain, the top three contributors for PM_1_ pollution were coal combustion, secondary inorganic aerosols, and industrial emissions [13]. As known, PM_1_ is a type of health-damaging particle mainly sourced from chemical combustion and secondary aerosol materials in China [12,14]. PM_1_ has a higher surface area–volume ratio which allows it to easily penetrate the lungs and potentially induce more adverse health effects compared to other PMs [10,15]. It was found that PM_1_ accounts for 80% of PM_2.5_ mass contents [12], indicating that PM_2.5_-related health effects are likely dependent on the presence of PM_1_. In fact, short-term PM_1_-dependent effects have been previously implicated in causing various diseases by assessing relative health risk metrics. This includes emergency ambulance calls, emergency department visits (EDV), and mortality [16,17,18,19,20,21,22]. Recently, studies have mostly focused on the implications of short-term PM_1_ exposure in hospital admissions related to cardiovascular diseases and strokes [23,24,25,26], less is understood about the impact on respiratory disease hospitalizations [27,28].

In addition, the associated health impact is accompanied by economic loss, estimated to account for 1% of GDP in 2060, with additional health expenditures having the largest impact [29]. This indicates the urgent need for further study to elucidate the underlying impact. Based on a time-series analysis in China, PM_2.5_ exposure was attributable to an increase of 362,007 hospital admission (HA) cases and 3.68 billion CNY expenditures [1], highlighting the health and economic burden of ambient PM pollution. However, the hospital expenditures associated with PM_1_ exposure remain unclear.

In the present study, we aimed to evaluate the health and economic burden related to short-term PM_1_ exposure in Beijing. We performed a time-series analysis to study the impact of short-term PM_1_ exposure on hospital admission for non-accidental, COPD, and respiratory infection (RI) diseases from 2015 to 2017 among 16 districts of Beijing, China. We then estimated the attributable number of HA and the increase in expenditures to elucidate the potential socio-economic burden of ambient PM_1_ pollution. The study presented here may shed light on PM_1_-associated health effects and hospital expenses, which aids in air pollution mitigation.

## 2. Materials and Methods

This study aimed to investigate the association of ambient PM_1_ exposure with hospital admissions (HA) and total hospital expenses for the three cause-specific diseases as follows: all non-accidental causes (all-cause), respiratory infections (RI), and chronic obstructive pulmonary disease (COPD) in Beijing, China. Air pollutant and health data of sixteen administrative districts in Beijing during the study period, from 1 January 2015 to 31 December 2017, were used for the present study.

### 2.1. Air Pollutant and Health Data Preparation

Four sets of data were collected for each of the sixteen districts in Beijing, including daily air pollutant concentration, daily meteorological data, daily HA, and daily total hospital expense. The process of data collection and preparation has been described in previous studies [10,22]. Briefly, we specifically requested hourly ambient PM_1_ levels from four stations, which are part of the Atmosphere Watch Network in Beijing [30,31,32]. The locations of these four stations are identified by their official station IDs, which are #54398, #54433, #54499, and #54594, as illustrated in our prior study [22]. In this paper, we calculated the average PM_1_ data from these four stations to represent the daily PM_1_ levels for Beijing, a methodology commonly employed previously [18,19]. Temperature and humidity levels were obtained from the China Meteorological Data Service Centre (http://data.cma.cn/) (Accessed on 1 March 2024) [10,33]. Additionally, daily HA and hospital expense data were obtained from the Beijing Municipal Health Big Data and Policy Research Center (http://www.phic.org.cn/) (Accessed on 1 March 2024) and categorized using the Tenth Revision of the *International Classification of Diseases* codes for all-cause diseases (codes A00–R99), RI (codes J00–J98), and COPD (codes J41–J44) [10].

### 2.2. Determination of PM_1_-Associated Effects on Daily HA

We conducted a two-stage approach to elucidate the association between PM_1_ exposure and daily HA risk of all-cause diseases, COPD, and RI in Beijing, in line with previous studies [1,22]. Briefly, in the first stage, time-series analyses using generalized linear models [34,35] were performed to assess PM_1_-associated effects on daily HA for non-accidental diseases, COPD, and RI in each district of Beijing. Daily HAs (*y*) were estimated assuming a Poisson regression model [34,35], where *μ* is the parameter representing the expected number of HAs in a specific district of Beijing on a given day. The concentration of PM_1_ (*P*) within a three-day moving average was used as the attribute variable in each district. Given that previous studies typically showed significant PM-associated effects on health variables at lag0–2 days (labelled as lag0–2), district analyses of lag0–2 PM_1_-associated relative risks for daily HA in Beijing were performed. In order to control for potential variations, other factors were considered in the model, including the following: (1) the variable “day of the week” (*DoW*) to account for possible variations within a week [36]; (2) a natural spline smoothing function for calendar day with 7 degrees of freedom per year (*Yrdf*) to control long-term temporal trends of HA risk [10]; (3) a natural spline smoothing function for temperature (*Temp*) and relative humidity (*H*) with six degrees of freedom for a three-day moving average to exclude potential non-linear and delayed impacts of meteorological conditions on HA risk [18,27]. The model is expressed as below:

(1) y~Poisson(μ)

(2) $$\log\left(\mu \right)\sim P+DoW+Yrdf+Temp+H$$

In addition to the lag0–2 estimations, delayed effects of PM_1_ exposure and HA risk were controlled by using single lag days (labelled as lag0, lag1 and lag2), four-day, and five-day moving average (labelled as lag0–3 and lag0–4).

Secondly, after obtaining the district-specific HA risk data in the first stage, meta-analysis was performed to assess the overall effect of PM_1_ exposure on each disease in Beijing. Using random-effect models, the lag0–2 effect data were estimated for overall HA risk, referred to as relative risk (*RR*) and represented as mean and related 95% confidence interval (95% CI) per 10 μg/m^3^ uptick of PM_1_ concentration. Percentage differences in HA risk with a 10 μg/m^3^ increase in PM_1_ concentration were then calculated as (*RR* − 1) * 100. A two-sided *p*-value < 0.05 was considered statistically significant.

### 2.3. Estimation of PM_1_-Associated Daily HA and Total Hospital Expense Change

Overall effect estimates of HA risk from the meta-analysis were utilized to calculate the attributable number (*AN*) and attributable fraction (*AF*) of daily HA and total hospital expenses due to PM_1_ exposure for all-cause diseases, based on previously demonstrated methods [1,37]. The above estimated *RR* at lag0–2 was first used to calculate *RR_i_* for each year *i*, as below:

(3) $${RR}_{i}=e^{\left(\beta \times \frac{P_{i}}{10}\right)}$$

where *P_i_* indicates PM_1_ concentration of year *i,* and *β* represents the estimated coefficient of daily HA per 10 μg/m^3^ uptick of PM_1_ concentration from the meta-analysis.

*AN_i_* of daily HA of each year *i* for Beijing was then calculated as [38,39]:

(4) $${AN}_{i}=\frac{{RR}_{i}-1}{{RR}_{i}}\times C_{i}$$

where *C_i_* indicates the total number of daily HA in year *i* for Beijing. Annual *AN_i_* values were then averaged to calculate three-year average *AN*. *AF_i_* of daily HA of each year *i* for Beijing were therefore obtained by dividing annual *AN_i_* by the sum of HA over three years in Beijing. Additionally, the upper and lower limit values of 95% CI of *β* were used to calculate the 95% CI values of *ANs* and *AFs* using the above formulas [40].

Furthermore, we calculated the *AN* of total hospital expense for all-cause diseases in each year *i* in Beijing (referred to as ${AN}_{ei})$, shown as below [41]:

(5) $${AN}_{ei}=E_{i}\times {AN}_{i}$$

where *E_i_* is the average expense of all all-cause HA in year *i* for Beijing.

AF of the total hospital expense for all-cause diseases in each year *i* in Beijing (labelled as *AF_ei_*) were calculated by dividing ${AN}_{ei}$ by the sum of total hospital expenses over three years in Beijing.

District-specific *AN* and *AF* of daily HA and total hospital expenses for all-causes diseases in Beijing were estimated, respectively, using *β*, *RR*, daily HA, and total hospital expenses of each specific district in Beijing.

All data analyses and graph plotting were performed using packages *dlnm* and *metafor* in the R software (version 4.2.1). All data were presented as mean ± 95% CI or as indicated. The figures are plotted using ArcGIS (version 10.0) and R software (version 4.2.1).

## 3. Results

Table 1 summarizes PM pollutant concentrations for Beijing from 2015 to 2017. PM_1_ concentration levels experienced a 24.01% annual average reduction throughout the study period in Beijing, with a three-year average of 48.23 ± 44.51 μg/m^3^. A similar decreasing temporal trend was also observed in the PM_2.5_ and PM_10_ pollutant levels.

**Figure 1:**
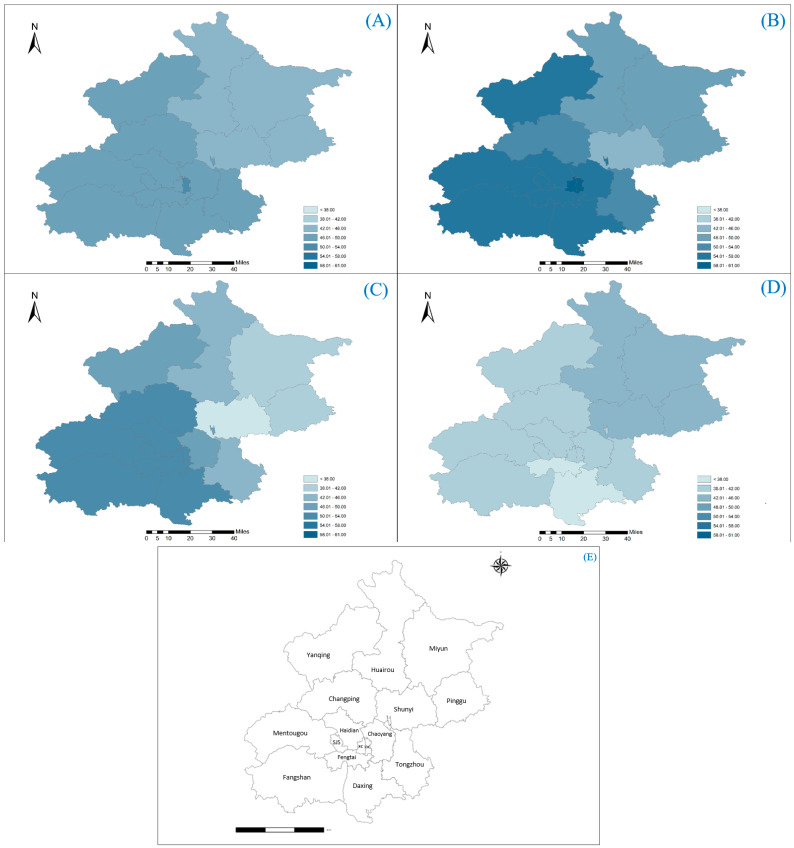
Heatmap illustrating the average PM_1_ concentrations (μg/m^3^) for each district in Beijing (**A**) average throughout 2015 to 2017; (**B**) in 2015; (**C**) in 2016; (**D**) in 2017. (**E**) Map indicating the locations of the 16 districts in Beijing. SJS, Shijingshan; XC, Xicheng; DC, Dongcheng.

Figure 1 shows PM_1_ concentration in each of the 16 districts of Beijing from 2015 to 2017. In general, the six urban districts (Dongcheng, Xicheng, Haidian, Chaoyang, Fengtai and Shijingshan) demonstrated higher three-year average PM_1_ concentration levels, compared to the other rural districts. The highest average PM_1_ level was reported in the Dongcheng district (50.93 ± 50.05 μg/m^3^) throughout the study period, which is in line with a previous study [42]. In Beijing, Dongcheng is one of the core districts. The highest concentration found in Dongcheng could be due to a combination of factors, including heavy traffic, industrial emissions, construction activities, and geographical location. Contrastingly, the lowest in Shunyi district (42.91 ± 38.78 μg/m^3^) is 15.74% lower than that in Dongcheng.

The daily average HA of all-cause, RI, and COPD patients are also summarized in Table 1. In total, 5,847,285 cases of all-cause, 125,772 cases of RI, and 86,597 cases of COPD HA were recorded, corresponding to a daily average HA of 333 ± 369 cases for all-cause, 7 ± 6 cases for RI, and 5 ± 5 cases for COPD over the study period in Beijing. Interestingly, the daily average HA for all-cause experienced an increasing temporal trend during 2015–2017, which raised by 19.73% in 2017 (358 ± 286 cases), compared to that of 2015 (299 ± 241 cases). Moreover, the total hospital expenses for all-cause reached 100,535 million CNY from 2015 to 2017 in Beijing, with a daily average of 5.73 ± 5.38 million CNY (Table 1).

**Figure 2:**
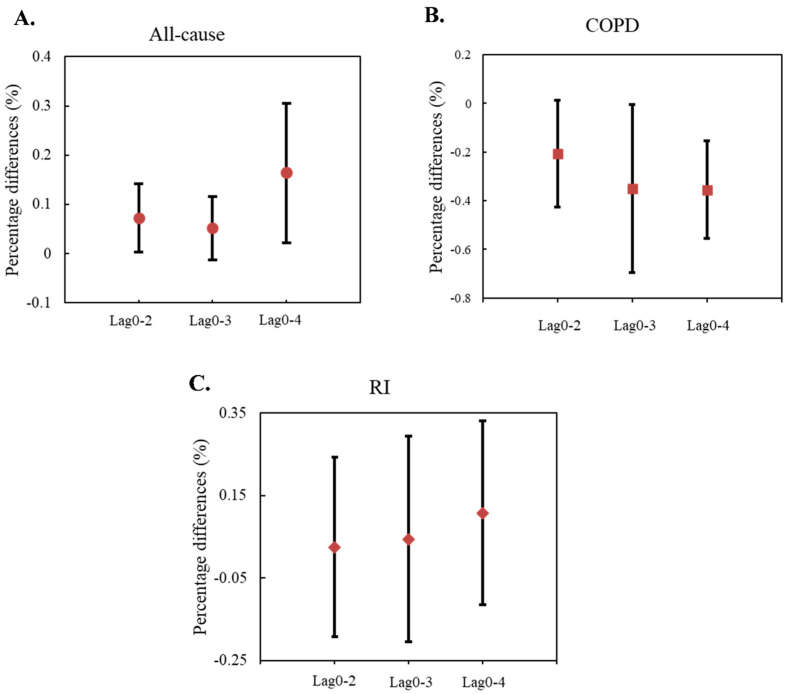
The percentage differences in hospital admissions per 10 μg/m^3^ uptick of PM_1_ exposure at different days of moving average during 2015–2017 in Beijing for (**A**) all-cause, (**B**) COPD, and (**C**) RI diseases.

**Figure 3:**
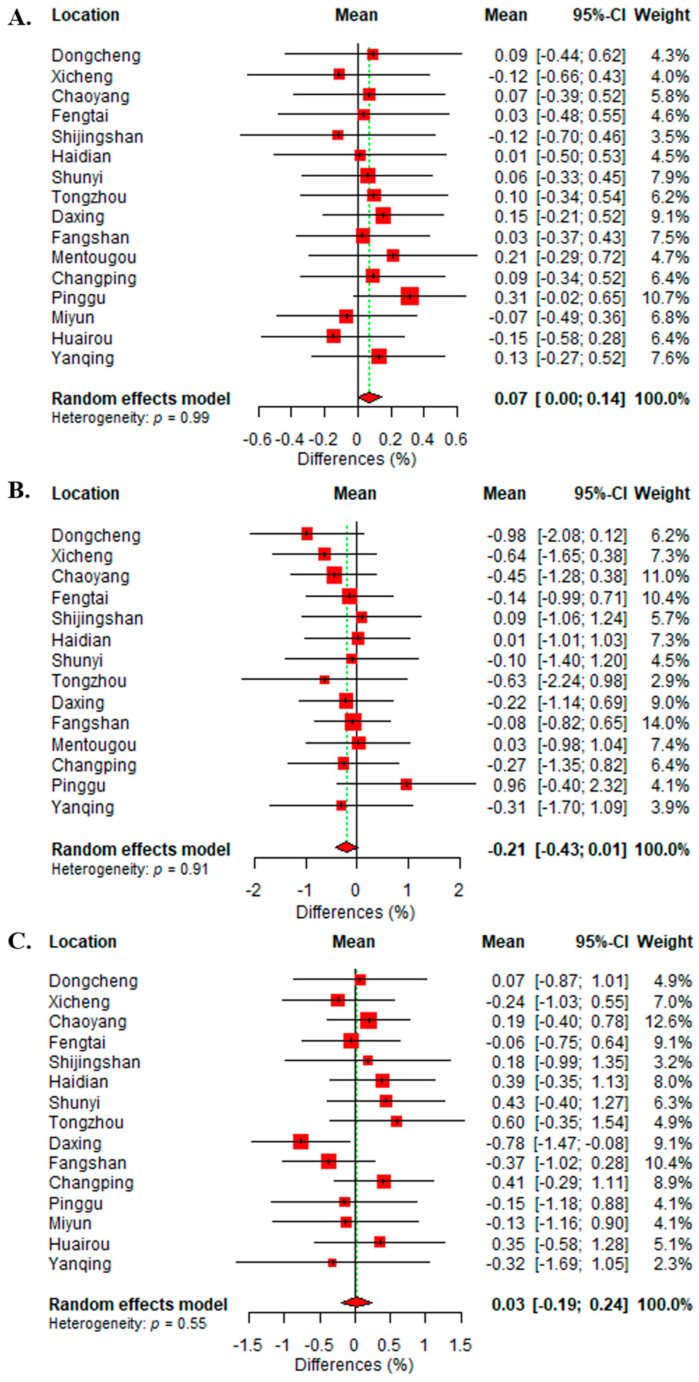
The forest plot for percentage differences of hospital admissions per 10 μg/m^3^ uptick of PM_1_ exposure during 2015–2017 in each district at lag0–2 days for (**A**) all-cause, (**B**) COPD, and (**C**) RI diseases.

Pooled estimations of PM_1_-associated effects on daily HA at different lag days during the study period in Beijing are illustrated in Figure 2. Percentage increases in HA per each 10 μg/m^3^ uptick in PM_1_ concentration are significant for all-cause admissions at lag0–2 and lag0–4 days. However, COPD and RI admissions failed to demonstrate significant PM_1_-associated risk increases. As illustrated in Figure 3, a 10 μg/m^3^ rise in PM_1_ concentration is associated with a 0.07% (95% CI: [0, 0.14%]) increase in daily HA for all-cause during 2015–2017. A total of 12 out of the 16 districts, including 4 urban and 8 rural districts, had significant PM_1_-associated effects on all-cause admissions during the study period. The strongest increment was seen in Mentougou district, corresponding to a 0.21% [−0.29, 0.72%] increase in daily HA for all-cause, and the lowest was observed in Fangshan district (0.03%, [−0.37, 0.43%]), although this was not statistically significant. The present study did not demonstrate a significant association between other dose metrics (for example, the PM_2.5_ and PM_10_) and all-cause admissions.

We then proceeded with the all-cause lag0–2 data for the estimation of HA and total hospital expenses attributable to ambient PM_1_ in Beijing during 2015–2017 (Table 2). The percentage increase in all-cause HA associated with ambient PM_1_ exposure decreased over time by years, with an average percentage of 0.34% [0.02, 0.67%] from 2015 to 2017 in Beijing. This is estimated as a daily average of 6644 [351, 12,917] admissions attributable to ambient PM_1_, corresponding to being 0.11% [0.01, 0.22%] related to PM_1_ exposure within the all-cause disease group. As for the attributable hospital expense estimations for all-cause diseases (Table 2), a three-year average number of 106 [5.6, 207] million CNY is estimated to be associated with ambient PM_1_ in Beijing during the study period. This is represented by a fraction of 0.32% [0.02, 0.62%] attributable to PM_1_ exposure. In particular, district analysis (shown in Table 3) reveals the strongest PM_1_-associated effect on all-cause associated HA and total hospital expense increases in Chaoyang district. It is estimated that 3540 [187, 6881] daily HA cases can be attributed to ambient PM_1_ pollution. This was found to result in a 63.68 [3.37, 123.79] million CNY increase in hospital spending, accounting for 18.37% of the city-wide attributable increase in hospital expenses.

## 4. Discussion

In this time-series analysis, we analyzed PM_1_-associated hospitalization risks for all-cause, COPD and RI in Beijing, China during 2015–2017. We demonstrated that ambient PM_1_ exposure was associated with an increase in all-cause HA but has no significant effect on COPD or RI-related hospitalization in Beijing. The average estimated hospital expenditure for non-accidental diseases attributable to PM_1_ exposure was 106 million CNY in Beijing, which accounts for a significant value of 0.32% (95% CI: 0.02, 0.62%) of the total estimated city expense. These findings indicate that short-term PM_1_ exposure could lead to remarkable health and economic burdens on Beijing, China.

Ambient exposure to PMs including PM_1_, PM_2.5_, and PM_10_, has long been implicated in various non-accidental clinical conditions, including RI, cardiovascular diseases [37,43,44], stroke [4], sleeping disorder [45], liver diseases [46], kidney functions [47], gene damage [48], reproductive disease [49] and obesity [50]. Recent studies have demonstrated significant epidemiological evidence of PM_1_-related health risks for all non-accidental diseases in China, including data on emergency department visits (EDV) [16,17,18,19,21,22] and mortality [10,37,51]. It was also reported that short-term PM_1_ exposure could lead to increased hospitalization related to ischemic stroke and cardiovascular diseases in China [23,24,25,26]. However, few studies have focused on changes in non-accidental and respiratory-related HA due to ambient PM_1_ exposure. Here, we report a significant 0.07% (95% CI: 0, 0.14%) increase in all-cause HA per 10 μg/m^3^ increase in PM_1_ concentration. Similar trends were recapitulated in all-cause EDVs and mortality associated with PM_1_ pollution in China. Wang et al. reported a 0.47% (95% CI: 0.35, 0.59%) increase in all-cause EDVs related to PM_1_ exposure in Beijing from 2016 to 2017 [22]. Similarly, Zhang et al. reported increases of 2.2% (95% CI: 1.8, 2.6%) and 1.7% (95% CI: 1.0, 2.4%) in all-cause EDVs per 10 μg/m^3^ increase in PM_1_ concentration in Guangzhou and Shenzhen, respectively, from 2015 to 2016 [18]. Compared to these prior studies, the stronger estimates were possibly due to lower PM_1_ level compared to Beijing and the use of different lag periods during statistical analysis [18,52]. Additionally, estimates could be affected by the fact that patients with acute diseases may not develop more serious conditions that require hospital admission and further treatments, even if they have visited the emergency department. Pooled analysis of all-cause mortality data in Beijing from 2014 to 2017 also showed a 0.19% (95% CI: 0.09, 0.28%) increase due to ambient PM_1_ exposure [10]. The reasons for such spatial heterogeneity and more significant estimates on PM_1_-related all-cause hospitalization risks could be due to different PM pollution sources, climate conditions including seasonal temperature and humidity, variable population vulnerability, access to healthcare and socioeconomical status [53].

Previous PM-related health analyses have mostly focused on PM_2.5_ and PM_10_. In 2019, a 35% reduction in Beijing’s PM concentration was reported [54]. We also observed a decreasing temporal trend in the levels of PM_1_, PM_2.5_, and PM_10_ pollutants from 2015 to 2017 in Beijing. Studies have shown that PM_1_ accounts for around 60% of the PM_10_ components and 80% of the PM_2.5_ particles across Chinese cities [12,55], indicating that ultrafine particles are the main compositions of PM mass pollutants. Previous estimations demonstrated that PM_1_, PM_2.5_, and PM_10_ exposure had similar or lower effects on all-cause hospitalization risks. Peng et al. reported that from 2014 to 2017, as PM_1_, PM_2.5_, and PM_10_ concentration increased by 10 μg/m^3^, non-accidental mortality elevated by 0.19% (95% CI: 0.09, 0.28%), 0.18% (95% CI: 0.08, 0.27%), and 0.17% (95% CI: 0.01, 0.24%), respectively [10]. PM_1_ is a health-damaging particle, which can easily penetrate the lungs and enter systemic circulation [15]. It was found that PM_1_ carries more toxic molecules, including metals and organic compounds, than other PMs, which can potentially induce adverse lung injury and genetic changes [14,56,57]. Compared to other PMs, PM_1_ triggers more significant pro-inflammatory responses and oxidative stress [15,58]. Given the implications of PM_2.5_ and PM_10_ in various respiratory and cardiovascular diseases [5,9,57], it is likely that the rising hospitalization risks associated with PM_2.5_ and more coarse PM particles are mainly attributed to their PM_1_ components.

Despite significant effects shown for all-cause PM_1_-related hospitalization risks, HAs associated with RI were not found to be significantly impacted by ambient PM_1_ exposure in Beijing from 2015 to 2017. In contrast, a case-crossover study in Shenzhen reported that a 10 μg/m^3^ increase in PM_1_ exposure was associated with a 0.09% (95% CI: 0.04%, 0.14%) increase in RI-related HA during 2015–2016, with a stronger effect observed during the cold seasons [18]. Similar results were reported in the Beibu Gulf area of China, where 3.0% (95% CI: 2.7%, 3.2%) were found to be attributable to PM_1_ pollution from 2013 to 2016 [28]. This is possibly due to the composition of PM_1_ particles, and as the main source of PM_1_ in Southern China is mobile emission [10], which causes more severe effects on lung functions, compared to non-vehicle pollutants [59]. A study in Hanoi, Vietnam, also showed that the components of PM pollutants affect the degree of heath impairment [60]. The PM_1_-associated increase in RI-related HA lost its significance after adjusting for NO_2_ as a confounding variable during the time-stratified case-crossover analysis [60]. Additionally, short-term exposure to PM_1_ was found to increase the risk of acute respiratory conditions, as evident by the significant correlation between PM_1_ exposure and respiratory HA during 2007 to 2012 in Beijing [61], and the PM_1_-related increase in respiratory EDVs in Guangzhou [16] and Beijing [22]. However, a meta-analysis of hospitalization, EDV and prevalence data from China, Vietnam, and America published between 2004 and 2021 showed that PM_1_ exposure had no significant association with the increase in RI hospitalization risks [17]. Therefore, there is still a lack of understanding regarding how PM_1_ exposure contributes to the development of respiratory diseases and the time it takes for disease progression, which may also explain the inconsistency of results reported here.

As one of the most common chronic respiratory diseases, we found that the hospitalization risk of COPD was not significantly associated with ambient PM_1_ exposure, although exposure to ultrafine particles, including PM_1_, was significantly implicated in COPD mortality in Shanghai [20] and COPD HA in Shenzhen [18]. Mei et al. previously found that COPD incidence levels were not significantly correlated with an increase in PM level and were less easily impacted by fine PM particles, compared to other respiratory conditions such as asthma [62]. Liu et al. mentioned that COPD prevalence differs significantly among different cities in China, as COPD may be affected by various disease causes such as age, smoking, and exposure to biofuels and dusts [63,64]. We also noticed a large heterogeneity of HA among different districts in Beijing for non-accidental diseases, including COPD. This large variation was recapitulated in an estimation analysis for EDVs in Beijing during the same study period, possibly due to differences in population vulnerability between age groups [22]. It was reported that COPD is more prevalent in rural areas of China [63], and given that Beijing has one of the best-quality healthcare system in China, patients from rural areas frequently visit the hospitals. Therefore, given the variation of demographic vulnerability to PM pollution, the underlying mechanisms of the week association between PM_1_ exposure and RI and COPD remain to be investigated.

Health expenditure has been one of the largest socio-economic burdens worldwide. It was reported that the government health expenditure in China experienced a 3-fold increase from 2008 to 2017 [65]. PM pollution has been recognized as one of the leading environmental causes for socio-economic burden globally [66]. Previous studies have mostly focused on the effect of PM_2.5_ pollution on healthcare expenditure related to non-accidental diseases. It was reported that PM_2.5_-asscoiated mortality may result in 101.39 billion US dollar, approximately 0.91% of China’s total GDP in 2016 [67]. A more recent nationwide study in China also found that an increase of 220 million CNY in healthcare expenditure was attributable to PM_2.5_-asscoiated hospitalization risks for lower RI during 2016 to 2017 [1]. Furthermore, a prior study found that PM_2.5_ exposure in China triggered an increase of 362,007 hospital cases [1]. However, to our knowledge, no study has yet estimated PM_1_-related hospitalization. Using Beijing as a case study, we found that non-accidental diseases attributable to PM_1_ exposure constitute 0.32% (95% CI: 0.02, 0.62%) of the total estimated city expense. Thus, reducing the pollutant levels of PM particles could largely avoid the economic loss due to the related health risk. Collectively, as industrialization and rapid modernization of cities worsens air quality, our findings could provide guidance for public policy makers and healthcare officials to reduce economic burden.

Nonetheless, the present study is limited by the lack of ground measurements of PM_1_ concentration. We only utilized ambient PM_1_ concentration from fixed-site monitoring stations, which failed to include individual heterogeneity regarding pollution exposure due to different outdoor activity times and habits and living conditions. We also did not analyze the confounding effects caused by other stimulants such as NO_2_, O_3_, and pollen exposure, which may not significantly shape the results [60].

## 5. Conclusions

In summary, as one of the world’s leading health threats, PM_1_ exposure is largely associated with various types of health conditions, including cardiorespiratory diseases. Using data on daily HA in Beijing during 2015–2017, our study provides evidence for PM_1_-associated impacts on all-cause HA increase by time-series analysis and meta-analysis. Our study conducted in Beijing during 2015–2017 showed that short-term PM_1_ exposure significantly increases the hospitalization risk of all-cause diseases. Accordingly, our data also showed that short-term PM_1_ exposure leads to additional hospital expenses in Beijing, thereby increasing health and economic loss. These findings highlight the necessity for effective air quality regulation and public health policies, particularly in developing countries such as China.

## Figures and Tables

**Table 1:** Summary statistics of air pollutants, cause-specific daily hospital admissions, total hospital expenses, and other variables for Beijing during 2015–2017.

Variables		Mean (SD)				Median (IQR)			
		2015	2016	2017	Average	2015	2016	2017	Average
Air Pollutant Concentration (μg/m^3^)	PM_1_	54.47 (49.08)	48.82 (43.58)	41.39 (39.38)	48.23 (44.51)	38.69 (50.20)	35.44 (47.29)	29.16 (37.28)	34.33 (45.54)
	PM_2.5_	71.66 (50.76)	63.18 (42.36)	56.07 (42.72)	63.63 (46.19)	58.96 (59.37)	54.26 (48.47)	44.31 (40.07)	52.23 (49.48)
	PM_10_	114.71 (85.67)	100.80 (68.20)	92.81 (76.42)	102.9 (77.62)	93.77 (90.59)	86.32 (75.58)	75.51 (62.01)	84.66 (76.53)
Temperature (°C)		12.7 (10.83)	12.57 (11.42)	12.97 (11.18)	12.75 (11.15)	14.56 (21.02)	14.54 (21.43)	13.80 (21.43)	14.34 (21.29)
Humidity (%)		55.57 (20.17)	54.44 (19.91)	52.24 (20.51)	54.08 (20.24)	56.21 (32.87)	54.37 (31.97)	50.30 (35.81)	53.94 (33.67)
Hospital Admissions (cases/day)	All-cause	299 (241)	343 (274)	358 (286)	333(269)	220 (290)	255 (326)	265 (337)	246 (319)
	RI	6 (5)	8 (6)	8 (5)	7 (6)	5 (7)	6 (8)	7 (8)	6 (7)
	COPD	4 (4)	5 (5)	5 (5)	5 (5)	3 (6)	4 (5)	4 (5)	4 (5)
Average Hospital Expense (All-cause, million CNY/day)		4.92 (4.58)	5.83 (5.41)	6.45 (5.96)	5.73 (5.38)	3.38 (4.64)	4.04 (5.56)	4.51 (6.21)	3.92 (5.53)

Abbreviations: SD, standard deviation; IQR, interquartile range.

**Table 2:** Attributable numbers and fractions of hospital admissions and total hospital expenses of all-cause diseases associated with ambient PM_1_ at lag0–2 in Beijing during 2015–2017.

Variables	Year	Percentage Differences in % (95% CI)	Attributable Number (95% CI)	Attributable Fraction in % (95% CI)
Hospital admissions (cases/day)	2015	0.38 (0.02, 0.75)	6697 (354, 13 016)	0.11 (0.01, 0.22)
	2016	0.34 (0.02, 0.67)	6856 (363, 13 328)	0.12 (0.01, 0.23)
	2017	0.31 (0.02, 0.67)	6382 (338, 12 408)	0.11 (0.01, 0.21)
	Average	0.34 (0.02, 0.67)	6644 (351, 12 917)	0.11 (0.01, 0.22)
Total hospital expense (million CNY)	2015		103 (5.4, 200)	0.36 (0.02, 0.70)
	2016		109 (5.7, 211)	0.32 (0.02, 0.62)
	2017		107 (5.7, 209)	0.28 (0.02, 0.55)
	Average		106 (5.6, 207)	0.32 (0.02, 0.62)

**Table 3:** Attributable numbers and fractions of hospital admissions and hospital expenses of all-cause diseases associated with ambient PM_1_ at lag0–2 of each district in Beijing during 2015–2017.

District	Hospital Admissions (Cases/Day)			Total Hospital Expense (Million CNY)	
	Percentage Differences in % (95% CI)	Attributable Number (95% CI)	Attributable Fraction in % (95% CI)	Attributable Number (95% CI)	Attributable Fraction in % (95% CI)
Changping	0.36 (0.02, 0.69)	1444 (76, 2807)	0.35 (0.02, 0.69)	21.64 (1.14, 42.07)	0.34 (0.02, 0.65)
Chaoyang	0.36 (0.02, 0.7)	3540 (187, 6881)	0.36 (0.02, 0.7)	63.68 (3.37, 123.79)	0.35 (0.02, 0.68)
Daxing	0.36 (0.02, 0.71)	1736 (92, 3375)	0.36 (0.02, 0.7)	22.67 (1.2, 44.07)	0.36 (0.02, 0.69)
Dongcheng	0.37 (0.02, 0.72)	1195 (63, 2323)	0.37 (0.02, 0.72)	23.62 (1.25, 45.9)	0.36 (0.02, 0.71)
Fangshan	0.36 (0.02, 0.7)	1411 (75, 2742)	0.36 (0.02, 0.69)	18.66 (0.99, 36.28)	0.35 (0.02, 0.67)
Fengtai	0.36 (0.02, 0.71)	2256 (119, 4386)	0.36 (0.02, 0.7)	41.14 (2.18, 79.98)	0.35 (0.02, 0.68)
Haidian	0.36 (0.02, 0.7)	2420 (128, 4703)	0.36 (0.02, 0.69)	45.76 (2.42, 88.95)	0.34 (0.02, 0.66)
Huairou	0.33 (0.02, 0.65)	412 (22, 801)	0.33 (0.02, 0.64)	5.56 (0.29, 10.82)	0.32 (0.02, 0.63)
Mentougou	0.36 (0.02, 0.7)	492 (26, 956)	0.36 (0.02, 0.7)	9.22 (0.49, 17.92)	0.35 (0.02, 0.68)
Miyun	0.33 (0.02, 0.64)	486 (26, 945)	0.33 (0.02, 0.63)	6.74 (0.36, 13.11)	0.32 (0.02, 0.62)
Pinggu	0.33 (0.02, 0.64)	585 (31, 1137)	0.33 (0.02, 0.63)	6.69 (0.35, 13)	0.32 (0.02, 0.63)
Shijingshan	0.36 (0.02, 0.7)	762 (40, 1481)	0.36 (0.02, 0.69)	14.66 (0.78, 28.5)	0.35 (0.02, 0.67)
Shunyi	0.31 (0.02, 0.61)	855 (45, 1662)	0.31 (0.02, 0.6)	12.74 (0.67, 24.77)	0.3 (0.02, 0.59)
Tongzhou	0.34 (0.02, 0.67)	1145 (61, 2226)	0.34 (0.02, 0.66)	16.73 (0.88, 32.52)	0.33 (0.02, 0.63)
Xicheng	0.36 (0.02, 0.71)	1621 (86, 3151)	0.36 (0.02, 0.71)	32.88 (1.74, 63.91)	0.36 (0.02, 0.7)
Yanqing	0.36 (0.02, 0.69)	312 (17, 607)	0.35 (0.02, 0.69)	4.23 (0.22, 8.22)	0.35 (0.02, 0.67)

## Data Availability

The authors do have the right to share the data. Data will be available based on reasonable applications and request.
